# Occupational exposure to HIV among healthcare workers in PMTCT sites in Port Harcourt, Nigeria

**DOI:** 10.1186/s12889-020-08528-5

**Published:** 2020-04-06

**Authors:** Ndubuisi Akpuh, IkeOluwapo Ajayi, Ayo Adebowale, Hadejia Idris Suleiman, Patrick Nguku, Mahmood Dalhat, Elizabeth Adedire

**Affiliations:** 1 Nigeria Field Epidemiology and Laboratory Training Program (NFELTP), 50 Haile Selassie Street, Asokoro, Abuja, Nigeria; 2 Department of Epidemiology and Medical Statistics College of Medicine, University of Ibadan, Ibadan, Nigeria; 3 Department of Community Medicine, Ahmadu Bello University Zaria, Kaduna State Nigeria

**Keywords:** Occupational exposure, HIV, HealthCare workers, Private facility, Public facility, PMTCT, Port Harcourt, Rivers state, Nigeria

## Abstract

**Background:**

Rivers State is among the states with high HIV prevalence in Nigeria. Occupational exposure to HIV through blood or body fluids of HIV/AIDS patients is a recognised risk factor of HIV infections among healthcare workers. We identified the determinants of occupational exposures to HIV among healthcare workers in Prevention of Maternal to Child Transmission (PMTCT) sites within Port Harcourt metropolis in Rivers State.

**Methods:**

A descriptive cross-sectional study was conducted and multi-stage sampling technique was used to select 341 healthcare providers from 22 public and 22 private health facilities in PMTCT sites in Port Harcourt metropolis. The data collected were analysed using descriptive statistics, Chi-square and logistic regression models (*p-value = 0.05*).

**Results:**

Respondents’ mean age was 35.9 ± SD8.4 years, 270 (80.1%) and 171(50.7%) were females, and from public health facilities respectively. Prevalence of occupational exposure of healthcare workers to HIV in the past 12 months was 153 (45.0%), and 96 (63.3%) experienced such exposure more than once. Contacts with potentially infectious body fluid accounted for the largest proportion 51 (33.3%); followed by needle stick prick 49 (32.6%). About 189 (56.1%) had safety information at their disposal and this serves as a reminder on safety precautions. The likelihood of occupational exposure was significantly higher among doctors (AOR = 2.22, 95% C.I = 1.16–4.25,) but lower among environmental health workers (AOR = 0.10, 95% C.I = 0.02–0.46,) than nurses/midwives when other factors were included in the model.

**Conclusion:**

Occupational exposure to blood and body fluids remains a frequent occurrence among healthcare workers; highest among doctors in PMTCT sites in the study area. Provision of protective safety materials, training and enforcement of adherence to universal precaution strategies are highly recommended.

## Background

Human Immunodeficiency Syndrome (HIV/AIDS) is one of the infectious diseases that threaten human survival [1]. Nigeria has the second largest number of people living with HIV/AIDS (PLHA) in Africa accounting for 9.0% of the global burden with prevalence of 3.2%, and burden of 3.1 million people living with HIV. This burden impacts negatively on the health system [2]. Report of HIV research in Nigeria showed that Rivers State has the highest HIV prevalence (15.2%) among the states in the country (National AIDS Reproductive Health survey, 2014) [3], but the latest National HIV/AIDS indicator survey places her in the third position in prevalence amongst 36 states and Federal Capital Territory (Federal Ministry of Health,2019) [4]. Healthcare workers are exposed to infection-causing organisms, including HIV as a result of caring for patients in the health care settings and this often places them at risk of infection [5].

Healthcare workers have become infected with HIV in caring for HIV patients [5] through accidental exposure to body fluids and percutaneous injury (needle stick or cut with a sharp object), contact of mucous membrane, or contact of skin (especially when the exposed skin is chapped, abridged, or afflicted with dermatitis [5–8]. Unfortunately, unavailability of protective equipment and or healthcare workers’ refusal to use ‘the safety equipment where available increases the risk of occupational exposure [9].

This study focuses on how health care workers are at higher risk of occupational infection compared to other healthcare workers in non-HIV specialised facilities [10]. It also focuses on specialized HIV care centres such as Prevention of Maternal to Child Transmission of HIV (PMTCT) sites in public and private settings [11]. This is because PMTCT sites are designated sites for HIV patients, and the care they receive during labour and child delivery almost certainly entail revealing body fluids which can infect a health worker [12] *.* There is a paucity of information on occupational exposure in private health care settings, and in HIV designated care centres in the available literature.

The objective of this study is to determine the prevalence of occupational exposure to HIV infection among healthcare workers in PMTCT sites and the outcome will make for policy direction from an informed perspective.

## Methods

### Study sites

Port Harcourt metropolis in Rivers State has designated PMTCT health facilities of public and private ownership. The private and public health facilities offer comprehensive healthcare services to clients. All study sites are reputed for good volume of client turn out and had at least a representative of the healthcare worker of interest whose day to day activity requires their contact with an HIV patient or their body fluid.

An Interviewer Administered Questionnaire was used to assess the occupational exposure to HIV by asking about accidental splashes with patient’s body fluids or prick by sharps while carrying out their duty. Also, their risk perception to occupational exposure, the practice of standard precaution procedure as well as the use of personal protective equipment. The availability of safety protocol/regulation and method of waste disposal were also assessed.

### Study design

We conducted a cross-sectional health facility-based study.

### Study population

The study population was made up of healthcare workers such as doctors, nurses, laboratory scientists or technicians and environmental workers whose daily activities require caring for HIV infected patients.

### Inclusion criteria

Healthcare workers in the selected health facility who were included in this study are doctors, nurses and midwives, laboratory scientist or technicians and environmental workers who were on duty and present at the time of visit; and gave their consent.

### Exclusion criteria

Trainee healthcare workers, healthcare workers that had assumed administrative responsibility and those who are less than 6 months into posting at present workplace were excluded from the study.

### Sample size calculation

The sample size of 341 was calculated using the formula,

$$ \mathrm{n}=\frac{{Z^2}_{\alpha }\  pq}{d^2} $$

Where,

*n* = the minimum sample size.

Z_α_ = the standard normal deviate corresponding to level of significance of 5% = 1.96.

d = the desired level of precision, 0.05.

*p* = proportion of HCW exposed to needle stick injury in Northern Uganda = 0.28 [13]

q = 1- *p* = 0.72.

10% non-response rate brings *n* = 341.

### Sampling technique

Multiple stage sampling technique was used to select the study subjects.

**Stage 1:** Health Facility Selection: All 22 public facilities within the study area designated PMTCT sites were sampled and an additional 22 of identified 34 private health facilities were selected by simple random sampling.

**Stage2:** Selection of health care workers from Health Facility: The study sample size of 341 was divided equally among the 44 selected health facilities giving 6 participants per facility.

**Stage 3:** Selection of cadre participants:

At the facility, one health care worker was selected by balloting to represent each cadre of health workers among doctors, nurses/midwives, laboratory scientist or technicians and environmental workers. The remaining two health workers were selected randomly from the four cadres of healthcare workers in the facility. Where a facility had six health care workers, all were studied and where less the remaining is made up from another facility.

### Pre-testing of data collection tool

Pre-testing of the questionnaire was conducted on 30 healthcare workers working in five health institutions that were not selected for the study before the actual data collection. Modification of logical sequence, simplicity, and clarity of questionnaire was done using the findings at the pre-test.

### Data collection

Data was collected using a semi-structured Interviewer-administered Questionnaire. The questionnaire had three sections. Section A: socio-demographic characteristics of health care workers such as age, sex and occupation. Section B: Occupational risk exposure to HIV, and Section C: Determinant factors to occupational risk exposure to HIV infection. The questionnaire was partly developed by us and some variables adapted from previous similar published works [1].

### Statistical analysis

We used Epi info 7 and SPSS statistical software to analyse descriptive variables and logistic regression to identify the independent risk factors associated with occupational exposure to HIV among healthcare workers using *p*-value of < 0.05 as the level of significance.

## Results

Of the 337 health care providers interviewed, 171 (50.7%) were recruited from public health facilities while. Sixty-three (18.7%) respondents were doctors, 124 (36.8%) nurses, 52 (15.4%) laboratory scientists or technicians, and 98 (29.1%) were environmental health workers. Study participants were predominantly Christians (336, 99.7%).

The majority (243, 72.1%) of the respondents had completed tertiary education. One-third (125, 37.1%) had worked for 10 years or more, and 246 (73%) work for an average of 40 h or more. Seventy-eight (23.1%) of the respondents were between the ages of 20–29 years, 156 (46.3%) were between the ages of 30–39 years, 75 (22.3%) were between the ages of 40–49 years, 24 (7.1%) were between the ages of 50–59 years and 4(1.2%) were 60 years old and above. The mean age was 35.9 ± 8.4 years Table 1.

**Table 1:** Characteristics of healthcare workers on the prevalence of occupational exposure to HIV infection in PMTCT sites, Port Harcourt, Nigeria

Socio-demographics	N	Experienced occupational exposure	Percent
Health care provider in PMTCT sites	337	153	45.40
**Age (Years)**			
20–29	78	32	41.0
30–39	156	66	42.3
≥ 40	103	106.7	154.7
**Mean age ± SD (years)**		**35.89 ± 8.4**	
**Occupational cadre**			
Nurse/midwife	124	64	51.6
Environmental health worker	98	20	20.4
Doctor	63	44	69.8
Laboratory scientist/technician	52	25	48.1
**Educational qualification**			
At most Primary	23	5	23.8
Secondary	71	18	25.4
Tertiary	243	130	53.5
**Years of experience**			
< 10 years	212	89	42.0
≥ 10 years	125	64	51.2
**Facility type**			
Public	171	70	40.9
Private	166	83	50.0
**Average working hour per week**			
< 40 h	91	46	50.5
≥ 40 h	246	107	43.5

The data also depicts that the overall prevalence of occupational exposure to HIV infection among the studied health care providers in PMTCT sites in Rivers State was 45.4% (Table 2). Among health care workers in the public health facilities, the prevalence of occupational exposure to HIV infection was 40.9% compared to 50% reported by those who work in the private health facilities.

**Table 2:** Infection prevention and patient safety (IPPS) standards in PMTCT sites Port Harcourt, Nigeria

Variable	Frequency	Percent
**Health workers who trained on IPPS (*n*** **= 337)**		
Yes	250	74.2
No	87	25.8
**Health care workers who need training on IPPS(*n*** **= 337)**		
Yes	330	98
No	7	2
**Health care worker last training on IPPS (*n*** **= 250)**		
Less than a year	114	45.6
More than a year	136	54.4
**PPE available for Healthcare worker use at PMTCT facility (n = 337)**		
Yes	262	77.7
No	75	22.3
**PPE regularly supplied at facility (*n*** **= 262)**		
Yes	176	67.2
No	86	32.8
**PEP available at PMTCT facility (n = 337)**		
Yes	188	55.8
No	149	44.2
**Someone available to administer PEP at facility (*n*** **= 188)**		
Yes	176	93.6
No	12	6.4
**Health care workers with access to PEP at facility (*n*** **= 337)**		
Yes	183	54.3
No	154	45.7
**Presence of reporting system for occupational exposure(*n*** **= 337)**		
Yes	206	61.1
No	131	38.9

The prevalence of occupational exposure to HIV was as high as 69.8% among doctors and as low as 20.4% among environmental health workers. Higher prevalence was found among males (53.7%) than females (43.3%).

The prevalence increased with the level of education from 23.8% among those with, at most, secondary education to 53.5% among those with higher education. It was lower among those with less than 10 years working experience 89 (42%) compared to those with at least 10 years 64 (51.2%).In terms of the number of working hours in a week, the prevalence of occupational exposure was higher for health care workers who worked less than 40 h (50.5%) compared to those who worked 40 h or more (43.5%) in a week.

Age-specific prevalence revealed 15.7% among healthcare workers aged 40 years and 42.3% for those between the ages of 30–39 years then 41.0% for those between the ages of 20–29 years.

**Fig. 1:**
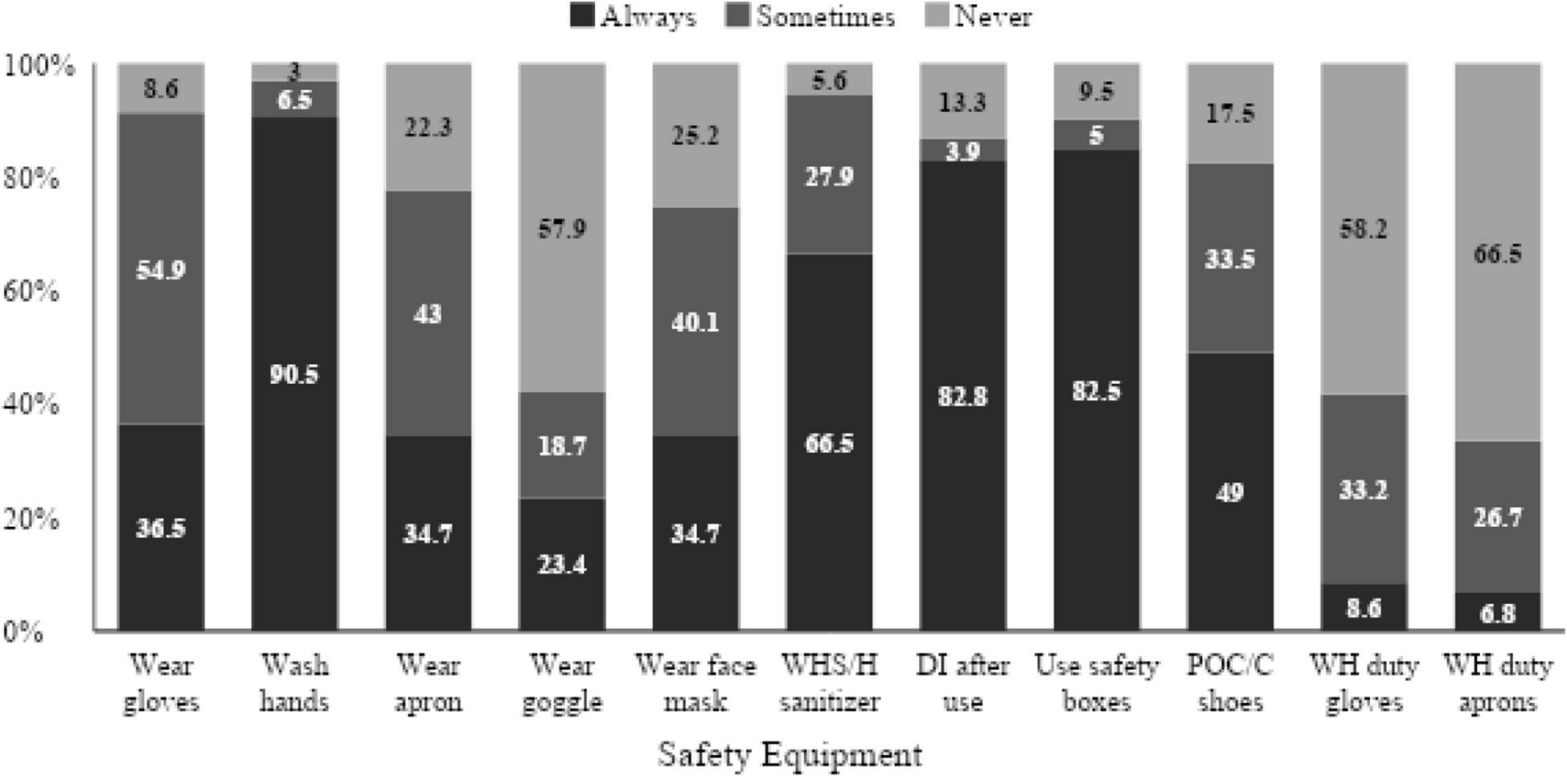
Practice of infection prevention procedures among healthcare workers in PMTCT sites Port Harcourt, Nigeria

Less than half of the health care workers, 123(36.5%) always wear hand gloves in the course of their duty, while 185(54.9) wear hand gloves sometimes and 29(8.6%) never wear hand gloves (Fig. 1).

Almost all 305 (90.5%) of the healthcare workers wash their hands all the time, 22(6.5%) sometime, and 10(3.0%) never wash hands while on duty. Regarding wiping hands with antiseptics, 224(66.5%) does it always while 94(27.9%) sometimes and 19(5.6) never. The face mask was always worn by 117(34.7%) and 85(25.2%) never did.

Up to 45(13.3%) of the respondents never decontaminated instruments immediately after use, 279(82.8%) always do, while 13(3.9) do some of the times.

A total of 278(82.5) respondents used safety boxes in the disposal of health waste, always as against 32(9.5) who never used a safety box. Regarding wearing personal protective equipment, only 168 (49.0%) wear protective shoes, 29(8.6%) use heavy duty gloves, and much less 23 (6.8%) wear aprons while at work.

**Fig. 2:**
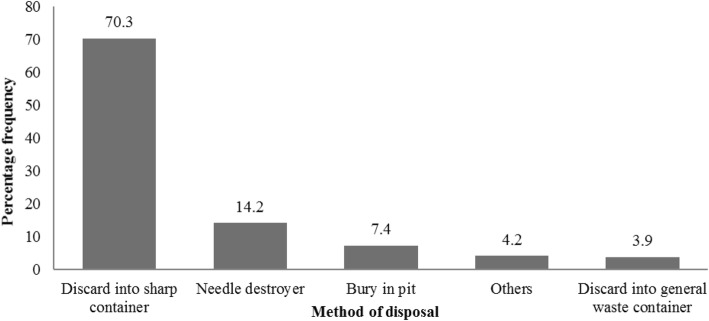
Sharps disposal methods in PMTCT sites, Port Harcourt, Nigeria

Figure 2 illustrates the disposal of sharps. Among the study participants, 237(70.3%) of respondents disposed of sharp equipment into containers set aside for sharps disposal. For some sharps like needles, 48 (14.2%) participants destroyed used needles in a needle destroyer, 25(7.4%) bury them in a health facility pit and 3.9% discard them into general waste containers. Only 4.2% dispose of sharps by other means that were not mentioned.

The main predictors of occupational exposure to HIV were career cadre. Doctors were more likely to have occupational exposure to HIV than another worker (AOR = 2.2, 95% C.I = 1.2–4.3, *p* < 0.05). On the other hand, environmental health workers appear to be protected when compared to nurses and midwives (AOR = 0.10, 95% C. I = 0.02–0.46, *p* < 0.01). Finally, there was no difference in risk of occupational exposure to HIV between health workers who work less than 40 h a week and those who work for at least 40 h (0.71(C. I = 0.44–1.12, *p* > 0.05) Table 3.

**Table 3:** Prevalence and determinants of occupational exposure to HIV infection among healthcare workers in PMTCT sites Port Harcourt, Nigeria

Factors assessed	Exposed (%)	Total	OR (CI)	AOR (CI)
**Age**				
20–29	32 (41.0)	78	1.65 (0.91–2.99)	1.78 (0.86–3.71)
30–39	66 (42.3)	156	1.56 (0.95–2.58)	1.80 (0.99–3.28)
40≥	55 (53.4)	103	1	1
**Occupational cadre**				
Doctor	44 (69.8)	63	2.17 (1.14–4.13) ^*c*^	2.22 (1.16–4.25) ^*c*^
Nurse/midwife	64 (51.6)	124	0.24 (0.13–0.44) ^*a*^	0.10 (0.02–0.46) ^*b*^
Laboratory scientist/technician	25 (48.1)	52	0.87 (0.45–1.66)	0.87 (0.45–1.68)
Environmental health worker	20 (20.4)	98	1	1
**Educational qualification**				
Completed at least Primary	5 (21.7)	23	0.24 (0.08–0.67) ^*b*^	2.85 (0.44–18.42)
Secondary	18 (25.4)	71	0.30 (0.16–0.53)	2.77 (0.57–13.29)
Tertiary	130 (53.5)	243	1	1
**Years of experience**				
< 10 years	89 (42.0)	212	1.45 (0.93–2.26)	0.94 (0.55–1.63)
≥ 10 years	64 (51.2)	125	1	1
**Facility type**				
Public	70 (40.9)	171	0.693 (0.45–1.07) ^d^	0.71 (0.44–1.12)
Private	83 (50.0)	166	1	1
**Average working hour per week**				
< 40 h	46 (50.5)	91	0.75 (0.47–1.22)	0.53 (0.30–0.92)
≥ 40 h	107 (43.5)	246	1	1

^*a*^*Significant at 0.1%;*^*b*^*Significant at 1.0%;*^*c*^*Significant at 5.0%;*^d^*Significant 10.0%*

## Discussion

Our findings indicate that about half of the healthcare workers had been exposed to HIV infection at their duty posts at one time or another in the past one year prior to this study. These findings are consistent and similar to the Bosnia/Herzegovinian, Cameroonian, Saudi Arabian studies on risk of occupational exposure conducted [11, 14, 15]. On the contrary, higher prevalence of occupational exposure to HIV infection among health care workers than the prevalence found in the current study has been reported by several other researchers in sub-Saharan Africa and other parts of the world [16]. But, our findings on prevalence of occupational exposure are above that obtained from similar studies conducted in Poland [10],Kuwait [17], South Korea [18] and Northern Nigeria [2]. This difference in prevalence of HIV observed in previous studies and our present study could be as a result of the difference in the study setting, study design and other methodological techniques. The differential in the level of training received by the health care workers in different study settings could also contribute to the variation.

It was also found that the prevalence of occupational exposure to HIV infection was higher among health care workers in private owned health facilities than their counterparts in the public health facilities; same finding was observed in a similar study conducted in Cameroon in 2018 [11]. Higher prevalence observed among health care workers in private health facilities might be due to the fact that private health facilities are profit-oriented. Private health facilities are likely to function with reduced manpower and possible extended work hours per week in order to maximize profit especially in the phase of prevailing economic challenges in Nigeria. In the phase of present economic realities, the private health care facilities may not be living up to the expectations of ensuring their healthcare workers’ adherence to standard health and safety regulations in terms of providing protective tools and training on standard infection prevention techniques. On the other hand, the public health facilities attract support from donor agencies which include procurement of safety tools for healthcare workers as well as periodic training. This support may not be readily available at the private facilities because of the more corporate profile and patient selective nature of private health facilities compared to public health facilities.

The risk of transmission of HIV infection from patient to healthcare worker has been shown by previous studies to be 0.3% in percutaneous exposure and 0.09% in muco-cutaneous exposure [19]. Contact with potentially infectious body fluid in both private and public facilities was found to be the commonest route of exposure to HIV infection in this study and it represents a substantive means of HIV transmission to a healthcare worker [20]. However, this finding is different from other studies where sharp injuries were found to be the commonest [15]. This outcome may be due to the fact that activities relating to dealing with sharps are more common among healthcare workers compared to dealing with activities that may cause splashes from body fluids. Other important risk factors to occupational exposures which were also found in this study and that could influence the experience of healthcare workers occupational exposure to HIV infection are lack of training on infection prevention and patient safety, unavailability and/or irregular supply of personal protective equipment, and inadequate post-exposure prophylaxis and shortages of personnel to administer post-exposure prophylaxis. The existence of poor infection prevention modalities exposes healthcare workers to HIV infection in PMTCT setting [21]. PMTCT sites at all times ought to function with the highest levels of infection prevention and control, given their nature as a specialized site where healthcare workers care for confirmed HIV positive patients and are at higher risk of contracting HIV compared to health workers in other non-HIV special-care health facilities either private or public.

In addition, we found that healthcare workers whose working hours were greater than 40 h were at higher risk of sustaining occupational exposure to HIV through percutaneous injuries and muco-cutaneous contaminations compared with other healthcare workers is not unique as similar study reported same in Mongolia [22]. This is because fatigue and exhaustion could lead to lack of concentration and can further predispose the healthcare worker to occupational exposure.

Some of the facilities studied had no established system for reporting occupational exposures. This was similar to the situation reported in areas of comparable resource setting [23]. The findings where a quarter of our study participants received no training on prevention of occupational exposure and almost all the participants wished to be trained on infection prevention and control is worrisome. These findings on deficient training on infection prevention are comparable to outcomes from similar studies in sub Saharan Africa [21]. The display of infection prevention and patient safety signs in health facilities are critical to attitudes of healthcare workers towards infection prevention and patient safety practices. Our research indicates that about fifty-percent of the studied PMTCT sites had guidelines for infection prevention and patient safety readily on display. This situation is a common practice among health facilities in other similar studies in Africa [24, 25].

The important predictors of occupational exposure found in this study were; cadre of health care workers and length of working hours [26]. Doctors were found to be at higher risk compared to other cadre of healthcare workers. This finding differs from findings from other studies where nurses were found to be at higher risk of occupational exposure [16, 27, 28]. High risk found among doctors in this study could be attributed to the fact that they are particularly involved in carrying out invasive procedures, doing veno-puncture and repair of episiotomy.

### Limitation of study

Potential limitation of study includes self-report rather than records review which is a more reliable means of evaluation of occupational exposure as healthcare workers are bound to report socially accepted information. The ability to recall occupational exposure in the past is also a limitation. We adjusted for this by training interviewers and limiting the period of exposure within one year. Result interpretations and findings from this study may not be generalised beyond PMTCT sites because of the cross-sectional nature of study design.

## Conclusion

The prevalence of occupational exposure to HIV infection among healthcare workers in PMTCT sites within Port Harcourt metropolis was high. Occupational exposure of health care workers to HIV infection is predicated upon the professional cadre of the health worker and high weekly working hours. In addition, the healthcare worker knowledge and adherence to the practice of infection prevention and patient safety is low. Also, despite the availability of protective equipment, all cadre of studied healthcare workers at one point attended to patients without protecting themselves. Some healthcare workers dispose of sharps instruments inappropriately. This implies that all health workers are at risk of HIV infection. Therefore, immediate training of healthcare workers on HIV infection prevention is recommended especially in privately owned PMTCT health facilities. There is also need to enforce the display of infection prevention guidelines and protocol in the PMCTC sites within the constant reach and visualization of healthcare workers. All these are necessary to ensure the safety and protection of healthcare workers thereby making the fight against HIV transmission holistic.

## Data Availability

The dataset used to produce this manuscript can be obtained from the corresponding author on reasonable request.

## Abbreviations

AIDS: Acquired Immune Deficiency Syndrome
AOR: Adjusted Odds Ratio
CI: Confidence Interval
HIV: Human Immune Virus
NFELTP: Nigeria Field Epidemiology and Laboratory Training Program
PLA: People Living with AIDS
PMTCT: Prevention of Maternal to Child Transmission of HIV
SD: Standard Deviation
SPSS: Special Package Statistical Software
